# Diabetestechnologie (Update 2023)

**DOI:** 10.1007/s00508-023-02165-9

**Published:** 2023-04-20

**Authors:** Ingrid Schütz-Fuhrmann, Birgit Rami-Merhar, Elke Fröhlich-Reiterer, Sabine E. Hofer, Martin Tauschmann, Julia K. Mader, Michael Resl, Alexandra Kautzky-Willer, Yvonne Winhofer-Stöckl, Markus Laimer, Sandra Zlamal-Fortunat, Raimund Weitgasser

**Affiliations:** 1 3. Medizinische Abteilung mit Stoffwechselerkrankungen und Nephrologie, Karl Landsteiner Institut für Endokrinologie und Stoffwechselerkrankungen, Klinik Hietzing, Wien, Österreich; 2 Universitätsklinik für Kinder- und Jugendheilkunde, Medizinische Universität Wien, Wien, Österreich; 3 Universitätsklinik für Kinder- und Jugendheilkunde, Medizinische Universität Graz, Graz, Österreich; 4 Department für Pädiatrie 1, Medizinische Universität Innsbruck, Innsbruck, Österreich; 5 Klinische Abteilung für Endokrinologie und Diabetologie, Universitätsklinik für Innere Medizin, Medizinische Universität Graz, Graz, Österreich; 6 Abteilung für Innere Medizin I, Konventhospital der Barmherzigen Brüder Linz, Linz, Österreich; 7 Klinische Abteilung für Endokrinologie und Stoffwechsel, Universitätsklinik für Innere Medizin III, Medizinische Universität Wien, Wien, Österreich; 8 Universitätsklinik für Diabetologie, Endokrinologie, Ernährungsmedizin und Metabolismus (UDEM), Universitätsspital Bern, Inselspital, Bern, Schweiz; 9 Abteilung für Innere Medizin und Gastroenterologie, Hepatologie, Endokrinologie, Rheumatologie und Nephrologie, Klinikum Klagenfurt am Wörthersee, Klagenfurt, Österreich; 10 Kompetenzzentrum Diabetes, Privatklinik Wehrle Diakonissen, Salzburg, Österreich

**Keywords:** Diabetes Technologie, Insulinpumpentherapie, Kontinuierliche Glukose Messung, Automatisierte Insulin Abgabe Systeme, Closed-Loop, Künstliches Pankreas, Mobile Apps, Telemedizin, Diabetes technology, Insulin pump therapy, CGM, Threshold-suspension, AID, Closed-loop, Artificial pancreas, Mobile apps, Telemedicine

## Abstract

Diese Leitlinie repräsentiert die Empfehlungen der Österreichischen Diabetes Gesellschaft (ÖDG) zur Nutzung von Diabetes-Technologie (Insulinpumpentherapie; kontinuierliche Glukosemesssysteme, CGM; Hybrid Closed Loop Systeme, HCL; Automated Insulin Delivery Systeme, AID, Diabetes-Apps) und den Zugang zu diesen technologischen Innovationen für Menschen mit Diabetes mellitus. Die Leitlinie wurde basierend auf aktueller wissenschaftlicher Evidenz erstellt.

## Abstract (en)

This Guideline represents the recommendations of the Austrian Diabetes Association (ÖDG) on the use of diabetes technology (insulin pump therapy; continuous glucose monitoring, CGM; hybrid closed-loop systems, HCL; diabetes apps) and access to these technological innovations for people with diabetes mellitus based on current scientific evidence.

## Grundlagen

### Insulinpumpen und kontinuierliche Glukosemessung

#### Einleitung

Neben der Entwicklung von neuen Insulinen mit vorteilhaften Wirkprofilen und innovativen Medikamenten haben Menschen mit Diabetes mellitus über die letzten drei Jahrzehnte vor allem auch von Fortschritten und Innovationen im Bereich der Diabetestechnologie profitiert. Insulinpumpen zur kontinuierlichen Insulinabgabe und Sensoren zur Glukosemessung haben sich sowohl im pädiatrischen Bereich als auch in der Betreuung von Erwachsenen im Vergleich zur Basis-Bolus-Therapie mittels Insulin-Pens und kapillären Blutzuckermessungen als zielführend und effektiv erwiesen [1–7].

## Therapieziele werden auf Technologie ausgerichtet

**Figure 1:**
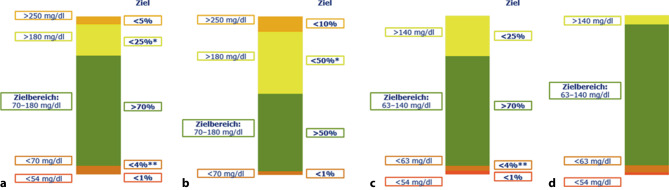

**Figure 2:**
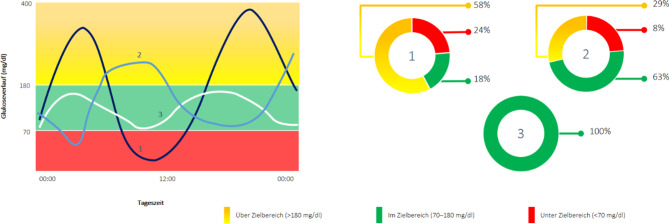

Der etablierten Messgröße HbA1c liegt ein Glukose-Mittelwert über zwei bis drei Monate zugrunde und gilt als gutes Maß für die Hyperglykämie, nicht jedoch für Hypoglykämien und Glukoseschwankungen. Letztere werden in Analogie zur Hyperglykämie ursächlich für mikrovaskuläre Spätkomplikationen gesehen. Durch die immer größere Verbreitung von CGM-Systemen sowohl bei Benutzer:innen von Insulin-Pumpen als auch Insulin-Pens stehen deutlich mehr Daten zur Verfügung als bei der herkömmlichen kapillären Blutzuckermessung. Der Begriff „Time In Range“ (TIR = Zeit im Zielbereich), d. h. der Prozentsatz an Zeit mit Sensorglukosewerten im Bereich zwischen 70–180 mg/dl, hat in den letzten Jahren zunehmend an Bedeutung gewonnen und ist zu einer neuen Messgröße zusätzlich zum HbA1c geworden [8, 9]. Mehr als 70 % eines Tages sollten im Zielbereich verbracht werden [8]. Eine TIR von 70 % entspricht dabei einem HbA1c-Wert von 7,0 % (53 mmol/mol) oder knapp darunter [8]. Hinsichtlich Vermeidung von Hypoglykämien wurde das Ziel weniger als 4 % des Tages unter 70 mg/dl und weniger als 1 % unter 54 mg/dl zu verbringen in Konsensus-Guidelines formuliert (Abb. 1; [8]). Die Zeit im Zielbereich korreliert mit dem HbA1c-Wert hinsichtlich des Folgeerkrankungsrisiko ([10]; Abb. 2).

## 1. Kontinuierliche Glukosemessung (CGM)

Unter kontinuierlicher Glukosemessung (continuous glucose monitoring, CGM) versteht man die Messung der Glukose im Subkutan-Gewebe alle 1–15 min mittels eines trans- oder subkutanen Sensors. Die Zeitverzögerung zum Blutzucker (Echtzeitglukose) beträgt je nach Messsystem ca. 10–20 min, wird aber durch die aktuelle Sensor-Messtechnologie nahezu ausgeglichen, sodass Sensormessungen neben Blutzuckermessungen immer häufiger direkt herangezogen werden können, um Therapieentscheidungen zu treffen. Die Messwerte werden mittels eines Empfängergerätes erfasst und gespeichert und die Daten mithilfe spezieller Softwareprogramme ausgewertet. CGM gilt als Methode der Wahl für die Glukosemessung bei Menschen mit Typ 1 Diabetes [11]. Dabei kann zwischen real time CGM (rtCGM) und intermittently scanned CGM (isCGM, Flash Glucose Monitoring) unterschieden werden. Neben der therapeutischen Anwendung von CGM kann diese professionell genutzt werden, um Glukoseverläufe retrospektiv zu analysieren und Therapieempfehlungen damit ermöglichen.

### 1.1. Real-Time-Monitoring CGM (rtCGM)

Kontinuierliche interstitielle Glukosemessung mit Anzeige der Werte gemeinsam mit einer Verlaufskurve der letzten Stunden auf einem Display am Lesegerät/Smartphone. Zusätzlich werden Trends mit Richtungspfeilen angezeigt. Alarme warnen bei Über- oder Unterschreiten definierter Grenzwerte. Die zugehörige Computer-App gibt statistische und graphische Auswertungen zum Glukoseverlauf über Tage bis Monate.

### 1.2. Intermittently scanned CGM (isCGM)

Kontinuierliche interstitielle Glukosemessung, wobei der/die Anwender:in nur Wert und Trend erhält, wenn er/sie einen Scan des Sensors mit dem Lesegerät/Smartphone durchführt. Es besteht dabei die Möglichkeit zur retrospektiven Analyse des Glukoseverlaufes sowie die aktuelle Trendangabe mittels Pfeilsystem, die aktuell zu erwartende Glukoseexkursion betreffend. Ein zweites System mit der Möglichkeit zur Alarmsetzung steht zur Verfügung. Auch dazu liefert die zugehörige Computer-App statistische und graphische Auswertungen zum Glukoseverlauf über Tage bis Monate.

## 2. Insulinpumpentherapie

Bei der Insulinpumpentherapie (oder continuous subcutaneous insulin infusion therapy, CSII) handelt es sich um eine Basis-Bolus-Therapie, bei der das Insulin subkutan mittels eines Katheters appliziert wird; sowohl kontinuierlich als sogenannte Basalrate (ersetzt das langwirksame Basalinsulin (‑Analogon)) und als Bolus (zum Essen und zur Korrektur) mittels eines schnellwirksamen Insulins (‑Analogon).

Sowohl die Vorteile der Insulinpumpentherapie (Dosierbarkeit 20-fach feiner als bei Pens, kontinuierliche Abgabe einer bis zu halbstündlich einstellbaren Basalrate, Unterstützung durch integrierten Bolurechner) als auch die Vorteile von Glukosesensoren (kein oder nur selten notwendiges blutiges Messen, „immer“ aktuelle Glukosewerte, Alarmfunktionen, Trendanzeige) prädestinieren beide Systeme für den Einsatz bei Menschen mit Diabetes mellitus Typ 1 und Personen mit anderen Diabetesformen, die eine komplexe Insulintherapie durchführen. Insbesondere im Kindes- und Jugendalter ist die Verwendung von Insulinpumpen und CGM-Systemen sehr weit verbreitet, und die Nutzungsrate beträgt bei Kleinkindern für Insulinpumpen und CGM-Systemen in Ländern mit optimaler Kostenerstattung > 90 % [12].

Durch die Verwendung von Insulinpumpen und Glukosesensoren kann es häufiger gelingen HbA1c-Werte und TIR weiter in den Normbereich zu verschieben, ohne dabei das Hypoglykämie-Risiko zu erhöhen [13].

### 2.1. Sensor-unterstützte Pumpentherapie (SUP), automatische prädiktive Hypoglykämie Abschaltung (PLGS)

In der Anwendung werden CGM-Systeme oft mit Insulinpumpen im Sinne einer sensorunterstützten Insulinpumpentherapie kombiniert. Die vorausschauende automatische Abschaltung der Insulinzufuhr bei drohender Hypoglykämie durch eine Pumpen-Sensor-Kombination ist in Österreich seit einigen Jahren verfügbar und hat sich vor allem in der Verringerung der Hypoglykämie-Häufigkeit und -Dauer bewährt [10, 14, 15].

### 2.2. Automated Insulin Delivery (AID) Systeme/Hybrid Closed Loop (HCL) Systeme

Diese Systeme verbinden derzeit die am weitesten fortgeschrittene Technologie. Bei komplexeren automatisierten Insulinpumpen/CGM-Systemen, auch als „Künstliche Bauchspeicheldrüse“, „Automated Insulin Delivery“ (AID) Systeme, „Hybrid-Closed-Loop-Systeme“ (HCL) bezeichnet, reguliert ein Algorithmus alle paar Minuten automatisch die Insulinzufuhr über die Pumpe unter Berücksichtigung der aktuellen und vergangenen sensor-generierten Glukoseverläufe. Im Vergleich zur Standardtherapie (Pumpe/Pen mit CGM), ist der Einsatz von AID-Systemen verbunden mit einer erhöhten TIR, sowie reduzierter Hyperglykämie und Hypoglykämie-Häufigkeit bei gleichzeitig moderater Reduktion der HbA1c-Werte [16, 17].

Eine Übersicht über die CE-zertifizierten Systeme [18–21] in der EU gibt Tab. 1. In Österreich sind aktuell (Stand 7/2022) nur Systeme der Firma Medtronic sowie eine Kombination aus Ypsopump, Dexcom 6 und CamAPS verfügbar bzw. durch Kostenübernahme der Krankenkassen gedeckt. Bei allen angeführten Systemen handelt es sich um Hybridlösungen, d. h. die basale Insulinabgabe wird durch den Algorithmus automatisch moduliert, den Mahlzeitenbolus gibt man manuell per Knopfdruck ab.

**Table 1:** 

	CamAPS FX	Diabeloop DBLG1/DBL4T	MiniMed 670G	MiniMed 770G	MiniMed 780G	T‑Slim X:2 mit Control IQ
Insulinpumpe	Dana R/S, Dana‑i od. Mylife YpsoPump	AccuChek Insight	MiniMed 670G	MiniMed 770G	MiniMed 780G	T‑Slim X:2
Glukosensor	DexCom G6	DexCom G6	Guardian 3	Guardian 3	Guardian 4	DexCom G6
Sensor-Funktionsdauer	10 Tage	10 Tage	7 Tage	7 Tage	7 Tage	10 Tage
Nötige Blutzucker-Kontrollen	Keine	Keine	Mindestens 4–6 ×/d	Mindestens 4–6 ×/d	Keine	Keine
Art des Algorithmus	MPC	MPC	PID	PID	PID mit Fuzzy Logic und MPC Anteil	MPC
Plattform des Algorithmus	Android Smartphone	Handgerät	In der Pumpe	In der Pumpe	In der Pumpe	In der Pumpe
Altersbeschränkung	> 1 Jahr (auch für Schwangere)	12–18 Jahre (DBL4T) > 18 Jahre (DBLG1)	> 7 Jahre	> 7 Jahre	> 7 Jahre	> 6 Jahre
Glukoseziel (mg/dl)	80–200	100–180	120	120	100, 110 oder 120	110
Automatische Korrekturbolusgabe	Nein	Ja	Nein	Nein	Ja	Ja
Möglichkeit der Datenverfügbarkeit	Automatisch, Diasend	Download, Diasend (Sensordaten Clarity)	Download, CareLink	Automatisch, CareLink	Automatisch, CareLink	Download, Diasend (Sensordaten Clarity)
Sonstiges	„Boost-Modus“	Variable „Aggressivität“	–	Per Handy anzusehen	Per Handy anzusehen	Nachtmodus
Temporäres Ziel erhöhen	„Ease off“/Aktivitätsmodus	Zen-Modus (20–40 mg/dl höher als aktuelles Ziel)	Temp. Ziel (150 mg/dl)	Temp. Ziel (150 mg/dl)	Temp. Ziel (150 mg/dl)	Aktivitätsmodus
Verfügbar in Österreich (08/22)	Ja (mit mylife YpsoPump)	Nein	Ja	Ja	Ja	Nein

*PID* „proportional integral derivative“, *MPC* „model predictive control“

Unterschiede bestehen in den Komponenten: je nach System kann es sein, dass trotz eines Glukosesensors zusätzlich zur Kalibrierung auch einzelne präprandiale kapilläre Glukosewerte nötig sind. Andere Sensoren kommen laut Herstellerangabe ohne kapilläre Glukosewerte aus. Manche Systeme können Glukosewerte per Bluetooth-Verbindung auf ein Smartphone übermitteln. Es gibt auch Systeme, bei denen der Steuerungsalgorithmus nicht in der Pumpe eingebaut ist, sondern sich auf einem externen Gerät bzw. als App auf einem Mobiltelefon befindet (Tab. 1).

## Einsatz von Diabetes Technologie in der Schwangerschaft

Einige RCTs haben demonstriert, dass die Verwendung von rtCGM während der Schwangerschaft die glykämische Kontrolle und das neonatale Outcome verbessern [22, 24]. Die Verwendung von isCGM bei Frauen mit Typ 1 Diabetes mit guter Einstellung unter MDI (multiple daily injection) zeigte eine signifikante Reduktion der TBR bei unveränderten HbA1c Werten [25]. In einer sekundären Analyse der CONCEPTT Studie hatten Kinder von Pumpen-Userinnen im Vergleich zu MDI-Userinnen eine höhere Wahrscheinlichkeit auf einer Intensivstation aufgenommen zu werden und eine neonatale Hypoglykämie, welche mit Glukose behandelt werden muss, zu erleiden. Die Lebensqualität wurde dagegen als besser beschrieben. Es zeigte sich im ersten Trimester kein HbA1c-Unterschied zwischen den Gruppen, wohl aber in der 34. SSW. Entscheidend für dieses Ergebnis war, dass Pumpen-Userinnen in der 24. SSW um 5 % weniger TIR (63 bis 140 mg/dl) verbrachten als MDI-Userinnen trotz vergleichbarer Ergebnisse im ersten und dritten Schwangerschaftsdrittel [23]. Eine weitere Analyse fand keinen Unterschied im Essverhalten zu den MDI-Userinnen. Es blieb die Tatsache, dass die Insulindosis nicht adäquat angepasst wurde [23] bzw. die unterschiedliche Kinetik des schnell-wirksamen Insulins in der Schwangerschaft berücksichtigt werden muss. Eine große Kohortenstudie berichtet über eine signifikante Verbesserung der täglichen Glukoseprofile, geringere Glukosevariabilität und geringere durchschnittliche Glukosewerte mit rtCGM verglichen mit der Blutzuckermessung bei Frauen mit Schwangerschaftsdiabetes. Die durchschnittlichen Glukosewerte waren signifikant mit dem Geburtsgewicht assoziiert und waren ein unabhängiger Risikofaktor für Präeklamsie und neonatales Outcome [26]. Rezentere Studien konnten keinen eindeutigen Vorteil von CGM zeigen, wobei in den Studien nicht zwischen Insulin behandelten und nicht Insulin behandelten Frauen unterschieden wurde [27, 29].

## Einsatz von Technologie im Kindes- und Jugendalter

Die Insulinpumpentherapie sollte allen Kindern und Jugendlichen jeglichen Alters empfohlen werden; bei den folgenden Indikationen aber jedenfalls eingesetzt werden [30, 31]:

- Kleinkinder (auch Säuglinge),
- Kinder und Jugendliche mit ausgeprägtem Dawn-Phänomen,
- Risiko für Hypoglykämien, rezidivierende Hypoglykämien, fehlende Hypoglykämiewahrnehmung, nächtliche Hypoglykämien,
- Nadelphobie,
- hohe glykämische Variabilität unabhängig vom HbA_1c_,
- bei Vorliegen von diabetischen Spätschäden (Retinopathie, Nephropathie),
- schwangere Jugendliche.

Alle derzeit erhältlichen und im Kindesalter zugelassenen Systeme (ab 1. Lebensjahr CamDiab, ab 7. Lj Medtronic 780G) sind Hybrid Closed Loop Systeme, das bedeutet, dass für die Mahlzeiten Boli abgegeben werden müssen. Die Anwendung dieser Systeme geht mit einer Steigerung der Zeit im Zielbereich, einer Reduktion der HbA1c Werte einher, ohne Steigerung von Hypoglykämien einher [32].

Eine gute metabolische Einstellung von Beginn an ist prognostisch essentiell [33–35] daher ist es notwendig, eine individualisierte, alters-adäquate Therapie anzuwenden, um eine hohe Therapiezufriedenheit und Compliance zu erreichen.

## Einsatz von Diabetestechnologie bei Menschen mit Typ 2 Diabetes

Aufgrund des progressiven Betazellversagens kann bei Menschen mit Diabetes mellitus Typ 2 eine Insulintherapie notwendig werden. Üblicherweise wird die Insulintherapie mit einer basal unterstützen oralen Therapie (BOT) begonnen. In einer RCT konnte eine signifikante HbA1c-Reduktion bei Menschen mit schlecht kontrolliertem Diabetes mellitus Typ 2 unter Einsatz von rtCGM gezeigt werden [36]. Obwohl in großen klinischen Studien mit isCGM nur minimale HbA1c-Verbesserungen in Populationen mit Typ 2 Diabetes auch mit einer intensiven Insulintherapie erreicht werden konnten, war die Anwendung von isCGM mit einer signifikanten Reduktion von Hypoglykämien sowie einer verbesserten Lebensqualität vergesellschaftet [37–40]. Eine verbesserte glykämische Kontrolle, eine verbesserte Gewichtskontrolle wie eine Änderung des Verhaltens konnte in einer limitierten Anzahl von Studien von großer Heterogenität für die Anwendung von CGM bei Menschen, die keine komplexe Insulintherapie durchführen, gezeigt werden [41, 42].

Die Insulinpumpentherapie kann bei Menschen mit Typ 2 Diabetes die HbA1c-Werte signifikant erniedrigen, wobei vor allem jene mit der schlechtesten glykämischen Kontrolle und den höchsten Insulindosen profitieren. Unter 8 % (64 mmol/mol) HbA1c ist der Effekt gering, wenn auch die Menschen persönlich zufriedener sind [43]. Seit dem Abschluss der OpT2mise Studie, einer RCT für Insulinpumpentherapie bei Menschen mit Diabetes mellitus Typ 2 sind viele neue medikamentöse Möglichkeiten entwickelt worden. Direkte Vergleiche oder Kombinationen von Pumpentherapie mit neuen Medikamenten finden sich in der Literatur nicht.

## Einsatz von Diabetestechnologie bei Menschen mit „anderen spezifischen Diabetesformen“

Die unter der Kategorie „andere spezifische Diabetesformen“ zusammengefassten Erkrankungen stellen pathophysiologisch und therapeutisch eine sehr heterogene Krankheitsgruppe dar. Die genauere Beschreibung der einzelnen Formen sowie deren Therapiemöglichkeiten werden in den ÖDG-Diabetesleitlinien an anderer Stelle beschrieben.

Grundsätzlich gibt es nur eine geringe Anzahl an wissenschaftlich relevanten Arbeiten, die den Einsatz von Technologien auch bei den häufigeren Formen wie z. B. dem pankreopriven Diabetes bisher untersucht haben. Es ist aber davon auszugehen, dass insbesondere absolut insulinabhängige Diabetesformen wie z. B. Menschen nach totaler Pankreatektomie auf Grund der instabilen Stoffwechsellage mit einer deutlich erhöhten Neigung zu Hypoglykämien besonders von der Nutzung der neuen Diabetestechnologien (CGM und AID Systemen) profitieren. Sofern der an Diabetes erkrankte Mensch die zur Nutzung dieser Technologien notwendigen Voraussetzungen erfüllt, soll der Einsatz dieser aus oben genannten Gründen großzügig erfolgen.

Für den Posttransplantations-Diabetestyp gibt es eine Studie mit geringer Fallzahl, die einen Vorteil für den Einsatz der Insulinpumpentherapie bei Nierentransplantierten Patienten im Vergleich zur Injektionstherapie zeigt [44].

Für den Diabetes im Rahmen einer zystischen Fibrose konnte in einem rezenten Review trotz dem Vorliegen von zumindest 14 Studien keine ausreichende Evidenz für die Nutzung von CGM Systemen nachgewiesen werden. Laut Autoren gibt es aber auch keine potenziellen negativen Effekte beim Einsatz dieser Technologien [45–47].

## Technologie als Grundlage für telemedizinische Betreuung

Alle Insulinpumpen, CGM-Systeme und HCL-Systeme können über Cloudbasierte Software ausgelesen werden bzw. werden automatisch in die entsprechende Cloud hochgeladen. Damit besteht die ideale technische Grundlage für eine telemedizinische Betreuung, die vor allem während der Sars-Cov‑2 Pandemie in vielen Diabetes-Zentren zu einer neuen Realität geworden ist [48, 49]. Auch über die Pandemie hinaus birgt die Telemedizin großes Potenzial in der Langzeitbetreuung von Menschen mit Diabetes mellitus. Um Telemedizin in die Versorgungsstruktur implementieren zu können bedarf es allerdings einer soliden Planung und Umsetzung unter Berücksichtigung rechtlicher und datenschutzrechtlicher Grundlagen.

## Vermittlung von Theorie und Praxis

Die Implementierung und Verwendung von Diabetes-Technologie muss fundiert vermittelt und trainiert werden [50]. Strukturierte formale Schulungsprogramme haben sich als effektiv im Sinne von verbesserter glykämischer Kontrolle, Akzeptanz und Zufriedenheit der Anwender:innen erwiesen [51–52]. Vor allem die standardisierte CGM-Analyse mittels AGP (Ambulantes Glukoseprofil) sollte von medizinischen Fachkräften im Diabetesbereich beherrscht und die Daten in Analogie zu Laborparametern (z. B. HbA1c) zur Verlaufskontrolle dokumentiert werden. Das multidisziplinäre Schulungs-Team sollte (pädiatrische) Diabetolog:innen, Diabetesberater:innen, Diätolog:innen, Psycholog:innen sowie Sozialarbeiter:innen umfassen. Um eine qualitativ hochwertige Versorgung zu gewährleisten sind kontinuierliche Fortbildungen der Diabetesteams unverzichtbarer Teil eines erfolgreichen Qualitätsmanagements.

## Therapie-Empfehlung der ÖDG für den Einsatz von Diabetestechnologie (CSII; CGM; HCL, Apps) inkl. Option zur Telemedizin bei Menschen mit Diabetes mellitus

### Evidenzklassen (EK)

- Ia: systematische Übersichtsarbeiten von Studien der Evidenzstufe Ib
- Ib: randomisierte vergleichende klinische Studien
- IIa: systematische Übersichtsarbeiten von Studien der Evidenzstufe IIb
- IIb: prospektive, insbesondere vergleichende Kohortenstudien
- III: retrospektive Studien
- IV: Evidenz außerhalb von Studien (Meinungen anerkannter Experten, Assoziationsbeobachtungen, pathophysiologische Überlegungen oder deskriptive Darstellungen, Berichte von Expertenkomitees, Konsensuskonferenzen, Einzelfallberichte)

1. *Der Routineeinsatz von CGM wird empfohlen bei*
  allen Menschen mit Diabetes, die eine intensive Insulintherapie durchführen, definiert als 3 oder mehr Insulininjektionen am Tag oder eine Insulinpumpe nutzen (Ia).
  neu diagnostiziertem Diabetes mellitus Typ 1 (IV).
  allen Menschen mit problematischen Hypoglykämien (häufige/schwere Hypoglykämien, Hypoglykämie-Wahrnehmungsstörung) (Ia).
  allen schwangeren Frauen mit Diabetes, die eine komplexe Insulintherapie durchführen, unabhängig vom Diabetestyp. Weiters Frauen mit Schwangerschaftsdiabetes, die eine Insulintherapie durchführen (Ib).
  rtCGM bzw. isCGM Systeme, die Alarme abgeben, sollen allen Menschen mit problematischen Hypoglykämien (häufige/schwere, nächtliche Hypoglykämien, Wahrnehmungsstörungen) empfohlen werden (Ia).
2. *Der Routineeinsatz von CGM kann empfohlen werden bei*
  Frauen, mit Schwangerschaftsdiabetes, welche keine Insulintherapie durchführen (IIb)
  Menschen mit Typ 2 Diabetes, die keine oder keine komplexe Insulintherapie durchführen (Ib).
3. *Der professionelle Einsatz von CGM zur Therapiefindung soll eingesetzt werden bei*
  neu diagnostiziertem Diabetes mellitus (IV).
  Menschen mit Diabetes und einer komplexen Insulintherapie, die CGM noch nicht nutzen (IV.)
  Menschen, mit problematischen Hypoglykämien, die CGM aber nicht in der Routine nutzen (IV).
  Menschen mit Typ 2 Diabetes ohne Insulin, welche dies als Schulungstool episodisch nutzen können (IV).
4. *Insulinpumpentherapie ohne CGM soll allen Menschen mit komplexer Insulintherapie zur Verfügung stehen, die aufgrund von Hautproblemen oder psychischer Überforderung (z.* *B. durch die CGM Daten) kein CGM nutzen können. Dabei soll der Blutzucker zumindest 4 mal täglich kontrolliert werden (Ib).*
5. *Die Insulinpumpentherapie kann bei Menschen mit Typ 2 Diabetes mit schlechter glykämischer Kontrolle und hohen Insulindosen eingesetzt werden (Ia).*
6. *Insulinpumpentherapie mit CGM oder SAP wird allen Menschen mit Diabetes, die eine komplexe Insulintherapie durchführen empfohlen, die sich aktiv dafür entscheiden oder ihre Therapieziele mit einer MDI nicht erreichen (Ia).*
7. *Insulinpumpentherapie mit PLGS wird Menschen mit Diabetes mit problematischen Hypoglykämien empfohlen (Ib).*
8. *„Automated Insulin Delivery“ (AID) Systeme (AID) „Hybrid Closed Loop“ (HCL) werden allen Menschen mit Typ 1 Diabetes empfohlen, um ihre TIR zu erhöhen, Hyper- und Hypoglykämien zu verhindern und ihre Lebensqualität zu erhöhen und sind bevorzugt einzusetzen. (Ib).*

## Interessenkonflikt

I. Schütz-Fuhrmann erhielt Vortrags- und Beratungshonorare von Abbott Diabetes Care, Medtronic, Dexcom, Insulet. B. Rami-Merhar hat in den letzten 36 Monaten von folgenden Firmen Honorare/Forschungsgelder erhalten: Eli Lilly, Medtronic, Insulet, Abbott, Sanofi. E. Fröhlich-Reiterer hat in den letzten 36 Monaten von folgenden Firmen Honorare/Forschungsgelder erhalten: Eli Lilly, Medtronic, Novo Nordisk, Roche. S.E. Hofer hat in den letzten 36 Monaten von folgenden Unternehmen, die auch fördernde Mitglieder der ÖDG sind, Forschungsunterstützungen und/oder Honorare erhalten: E. Lilly, Medtronic, Insulet, Dexcom und Ypsomed. M. Tauschmann hat in den letzten 36 Monaten von folgenden Firmen Vortrags- bzw. Beratungshonorare erhalten: Abbott Diabetes Care, Eli Lilly, Medtronic. J.K. Mader erhielt Vortragshonorare von AstraZeneca, Abbott Diabetes Care, Böhringer-Ingelheim, Eli Lilly, Medtronic Minimed, Menarini, Merck Sharp Dohme, Nintamed, Novo Nordisk, Roche Diabetes Care, Sanofi-Aventis, Servier, Wellion, Ypsomed. Advisory Board: Abbott Diabetes Care, Böhringer-Ingelheim, Eli Lilly, Medtronic Minimed, Novo Nordisk, Roche Diabetes Care, Sanofi-Aventis. Gesellschafterin decide Clinical Software GmbH. Forschungsgelder von A. Menarini Diagnostics, B. Braun, Metronom Health, Novo Nordisk, Profusa Inc., Roche Diabetes Care, Sanofi-Aventis. M. Resl hat Vortragshonorare und Honorare für Beratungstätigkeit von folgenden Firmen erhalten: Boehringer Ingelheim, sanofi-aventis, Novo Nordisk, Dexcom. A. Kautzky-Willer hat von folgenden Unternehmen, die auch fördernde Mitglieder der ÖDG sind, Forschungsunterstützungen und/oder Honorare erhalten: AstraZeneca, Amgen, Boehringer Ingelheim, El Lilly, Merck Sharp Dohme, Novartis, Novo Nordisk, Roche, sanofi-aventis, Servier, Takeda. Y. Winhofer-Stöckl hat von folgenden Unternehmen, die auch fördernde Mitglieder der ÖDG sind, Forschungsunterstützungen und/oder Honorare erhalten: Abbott, AstraZeneca, Amgen, Boehringer Ingelheim, Eli Lilly, Merck Sharp Dohme, Medtronic, Novartis, Novo Nordisk, Roche, Sanofi-Aventis. S. Zlamal-Fortunat hat in den letzten 36 Monaten Hororare für Vorträge und Advisory Boards von Novo Nordisk, Elli Lilly, Astra Zeneca, Boehringer Ingelheim, Medtronic, MSD, Daiicchi Sankyo und Novartis erhalten. R. Weitgasser erhielt Vortrags- und Beratungshonorare von Abbott Diabetes Care, Eli Lilly, Medtrust, Novo Nordisk, Roche Diabetes Care, Sanofi. M. Laimer gibt an, dass kein Interessenkonflikt besteht.
